# Mental skills in Serbian handball players: In relation to the position and gender of players

**DOI:** 10.3389/fpsyg.2022.960201

**Published:** 2022-08-15

**Authors:** Damjan Jakšić, Jovana Trbojević Jocić, Stefan Maričić, Bülent O. Miçooğullari, Damir Sekulić, Nikola Foretić, Antonino Bianco, Patrik Drid

**Affiliations:** ^1^Faculty of Sport and Physical Education, University of Novi Sad, Novi Sad, Serbia; ^2^Matica Srpska, Novi Sad, Serbia; ^3^Physical Education and Sport Department, Nevşehir Hacı Bektaş Veli University, Nevşehir, Turkey; ^4^Faculty of Kinesiology, University of Split, Split, Croatia; ^5^Sport and Exercise Sciences Research Unit, University of Palermo, Palermo, Italy

**Keywords:** Bull’s questionnaire, mental preparation, handball, gender, team position, mental skills

## Abstract

**Objectives:**

Despite the potential link between mental skills and athletic performance, little is done to examine handball players’ present level of mental skills concerning their performance. To begin with, the study has three folded aims; the first one is to examine the factor structure of Bull’s Mental Skills Questionnaire, which was developed in the United Kingdom to measure selected mental skill, of Serbian athlete population. The second aim is to determine gender differences in those mental skills, and the third aim is to determine differences between the playing positions in the mental skills of handball players to create a mental profile of Serbian handball players.

**Materials and methods:**

The sample consisted of 170 handball players, aged 14 to 39, who have played handball at the semi-elite, competitive-elite, and successful-elite level. The modified exploratory factor analysis was used to determine the latent dimensions of the Bull’s Mental Skills Questionnaire. For examining gender differences in the manifestation of mental skills Mann–Whitney *U* test was used.

**Results:**

Compared to the original structure of the questionnaire, which singles out seven factors of mental skills (imagery ability, mental preparation, self-confidence, anxiety and worry management, concentration ability, relaxation ability, and motivation), five factors were singled out in the sample of Serbian male and female handball players (anxiety and concentration management—α = 0.74; self-confidence—α = 0.75; relaxation ability—α = 0.66; mental preparation—α = 0.68, and imagery ability—α = 0.66). With these five subscales as dependent variables, results of the Mann–Whitney *U* test show that there are significant gender differences in variable anxiety and concentration management (*U* = 2893.5, *p* = 0.049) and relaxation ability (*U* = 2833.0, *p* = 0.031). Female handball players score higher on anxiety and concentration management and lower on relaxation ability. When playing positions are in question, results of Kruskal–Wallis‘s one-way analysis of variance, i.e., Mann–Whitney’s *post hoc* analysis, suggest that statistically significant differences were observed between wings and center backs and wings and goalkeepers in the imagery ability.

**Conclusion:**

The Bull’s Mental Skills Questionnaire in Serbian sample of handball players show satisfactory psychometric characteristics but has singled out five factors of mental skills compared to the original questionnaire.

## Introduction

With the development of modern sports, there was a need to further explore the processes, characteristics, and skills of successful athletes in relation to those who do not reach the elite level. The increasingly advanced physical dispositions of today’s athletes have indicated that at the top competitive level, the difference between victory and defeat sometimes depends on psychological factors. Among the elite athletes in various sports, the winners of Olympic medals, the following differentiating characteristics were singled out: personal dispositions (such as optimism, adaptive perfectionism, sports intelligence, etc.); psychological skills or states (self-confidence, concentration, an optimal level of arousal, etc.); cognitive and behavioral techniques which athletes use for the purpose of achieving desirable psychological states (goal setting, self-talk, imagination, etc.) (Gould and Maynard, 2009).

However, when discussing the psychological characteristics of successful athletes, the term mental toughness is most often used. This term has been used for more than 20 years, along with terms such as mental strength, mental preparation, mental skills, and psychological skills. The impossibility of defining a clear and precise construct that would refer to the mental profile of highly successful athletes is still one of the challenges of sports psychology. Namely, while some researchers believe that mental toughness is a personality trait, others argue that it is a skill subject to learning, which is relatively stable, but also in interaction with environmental impacts (Gucciardi et al., 2015). In this manner, mental toughness remains primarily a subjective concept, which is most often viewed as a set of beliefs and behaviors of athletes that help them to overcome and master training and competitions (Liew et al., 2019).

One of the first studies to study the mental toughness of athletes is based on the idea that environmental factors (such as parental behavior and relationship with parents, experiences while growing up, socio-cultural background, etc.) form the basis of mental toughness consisting of three categories: strong character, strong attitude, and strong mind (Bull et al., 2005). The environment influences the formation of a strong character through which strong attitudes are manifested, and as the final result, a strong mind appears as a key psychological characteristic of mental toughness (Petrović and Trbojević, 2020). The mental toughness observed in this manner can be manifested in situations of a reversal, critical moments, and situations that require endurance and involve risk management (Bull et al., 2005). In sports, especially in modern handball, where fast reaction, precision, and application of various motor movements are required, along with the quick assessment of the situation and decision-making, these situations are more than everyday occurrence in competitions. In these situations, the athletes have the task of maintaining their self-confidence in moments when the game and their achievement are not favorable, remaining focused during, e.g., shooting the seven-meter throw and regulating emotions under pressure, to endure to the end in a physically demanding match, and to take responsibility for their own actions and decisions. According to Bull and associates, the realization of these tasks is possible due to a strong mental mind.

One of the most current models of mental toughness, which is largely empirically confirmed (e.g., Gucciardi et al., 2009), is a model of Jones et al. (2007), who define mental toughness as innate or learned psychological acuity allowing an athlete to cope more successfully with the demands of the sport than its opponent. This psychological acuity contributes to an athlete being more successful than other athletes in determination, focus, self-confidence, and stress coping (Jones et al., 2002). This understanding and interpretation of mental toughness are one of the most accepted definitions, with critics noting that it also primarily refers to the description of what it allows athletes to do and not what it really is (Crust, 2007). To determine what mental skills make successful athletes, Jones et al. (2007) interviewed Olympic and world gold medalists in various sports. Based on the interviews, the authors singled out four groups of attributes of the mental toughness of successful athletes (Table 1). The categories set up in this manner show that there is one general dimension of mental toughness that refers to the general state of mind—a mindset and three dimensions that are characteristic for a certain period of time during sports. According to this model, an athlete uses specific psychological skills for a certain process in sports. Such isolated attributes of mental toughness are consistent with previous studies where the importance of self-confidence, attention, regulation of emotions, and anxiety is emphasized, with the use of techniques such as self-talk, goal setting, visualization, and imagination (Petrović and Trbojević, 2020).

**TABLE 1:** Attributes of mental toughness (Jones et al., 2007).

General attitude/mindset dimension	Training	Competition		Post-competition
Belief	Long term goals as the source of motivation	Belief	Staying focused	Handling failure
Focus	Controlling the environment	Regulating performance	Handling pressure	Handling success
	Pushing yourself to the limit	Awareness and control of thought and feelings	Controlling the environment	

Having in mind the diversity in understanding mental toughness, in this research, we integrate the previously mentioned understandings, and we start from the notion of mental skills, which we regard as a set of learned or innate beliefs, attitudes, emotions, and behaviors that are specific to the sports setting and affect the way an athlete approaches the sport and reacts to different situations of achievement and pressure to achieve success in sports.

One of the questionnaires that found its practical and empirical use in sport is Bull’s Mental Skills Questionnaire (Bull et al., 1996), which was developed in the United Kingdom to measure imagery ability (IA), mental preparation (MP), self-confidence (SC), anxiety and worry management (AWM), concentration ability (CA), relaxation ability (RA), and motivation (M) (Bull et al., 1996). Even though there is a number of tools (questionnaires) that test in some way athlete’s mental skills, Bull’s questionnaire has a more comprehended view of mental skills and has been validated in South Africa and the United Kingdom context (Edwards and Steyn, 2011; Edwards et al., 2011; Edwards and Edwards, 2012), as well as in Turkey (Miçooğullari et al., 2021). The questionnaire was used in different research questions, such as defining psychosocial skills as important for talent development (exp. Olszewski-Kubilius et al., 2019), the relation between mental skills and psychological well-being (exp. Edwards and Steyn, 2008); the relation between mental skills and bowling accuracy (exp. Khan and Mitra, 2020). But, most of these studies haven’t applied BMSQ in team sports such as handball. One of the studies that used BMSQ in team sports investigated if athletes from individual sports and athletes from team sports differed in mental skills (Demir et al., 2020) and showed that there were meaningful differences between sport types and mental skills, but there were no meaningful differences between gender and mental skills. Athletes engaged in team sports had higher scores on mental preparation and self-confidence (Demir et al., 2020). Handball is an indoor, collective, and highly dynamic sport involving complex and sudden changes that require quick reaction and a broad focus of attention to adequately assess the position of the opponents and teammates. Research on the mental skills of handball players is still in its infancy, and one recent study compared the mental skills of American football and handball players and found that football players achieve statistically higher scores on confidence, control, and total mental toughness than handball players and that football players achieve lower scores at constancy and anxiety levels compared to handball players (Ekmekçi and Miçooğulları, 2018).

As stated, research into the mental skills of top athletes is a current topic in sports and sports psychology. Regarding data relating to handball, a slightly smaller number of studies can be found that relate to the examination of the psychological characteristics of handball players. Concentration, anxiety management, self-confidence, and motivation have been singled out as specific psychological skills in handball (Thomas et al., 1999; Kiss and Balogha, 2019).

Research on gender differences in athletes’ mental skills shows inconsistent data. Namely, although some studies indicate significant gender differences in the degree of emotional regulation in favor of male handball players (e.g., Kristjánsdóttir et al., 2018), no gender differences in the degree of anxiety as a personality trait were found in the sample of Serbian male and female handball players, and male and female Serbian handball players were found to achieve average scores on anxiety as a personality trait (Jakšić et al., 2020). Some studies have found that female football players react and perceive more intense stress and that they experience less control in stressful situations (Kaiseler et al., 2012). Similarly, in the sample of junior female handball players of the Ukrainian national team, the result shows that female handball players achieve higher scores on competitive anxiety and are more prone to self-blame compared to male handball players (Ivaskevych et al., 2019). In the sample of the junior Hungarian handball team, the authors found that male handball players achieved significantly higher scores on Coping with Adversity and Freedom from worry, while female handball players scored higher scores on Goal Setting/Mental Preparation and Coachability (Ökrös et al., 2020). The results suggest that male handball players have fewer concerns about performance and that they have more adaptive coping mechanisms than female handball players, as well as that female handball players, have a more pronounced ability to set goals and use mental preparation as part of their sports experience. Also, compared to male handball players, female handball players follow the instructions of the coach to a greater extent and better cope with constructive criticism by coaches compared to male handball players.

When it comes to different playing positions in handball and mental skills, research shows that goalkeepers, wings, and playmakers react faster when selecting adequate figures than those in the pivot or back positions (Kiss and Balogha, 2019). On the other hand, one of the few studies that examined the differences in certain psychological characteristics of Hungarian junior male and female handball players in terms of age and the playing position found that playmakers score highest in terms of Ability to concentrate, Coping with Adversity, Peaking under Pressure, Goal Setting/Mental Preparation, Coachability which speaks to the high mental skills of the players in this position (Ökrös et al., 2020). Namely, the playmaker is the leader of the game and, therefore, must be able to change the focus of attention from a wide to narrow focus, stay calm in tense situations and develop an action. Wings achieve slightly higher scores on self-confidence and achievement motivation compared to other playing positions, while goalkeepers score the lowest scores on these characteristics.

One of the goals of this research is to examine the factor structure of Bull’s Mental Skills Questionnaire. The questionnaire has not been applied to a sample of Serbian handball players so far. Research conducted outside the Serbian population so far on this questionnaire shows a satisfactory factor structure and application to athletes (Edwards et al., 2014). Bull’s questionnaire was selected because it measures a wide range of mental skills which postulate the previously mentioned notions of mental toughness. The second goal of this research is to determine gender differences in the mental skills of handball players. The third goal of the study is to determine differences in relation to the playing positions in the mental skills of handball players in order to create a mental profile of Serbian handball players.

## Materials and methods

### Study design

We used a cross-sectional correlational design to collect self-report data on mental skills from Serbian handball players.

### Sample

The research includes male and female handball players of various competitive ranks. The sample consisted of 170 handball players; male players (*N* = 99) and female handball players (*N* = 71), aged 14–39 ($\bar{X}$ = 22.03), who have played handball on at the semi-elite, competitive-elite, and successful-elite level^1^ [according to Swann et al. (2015)] between 1 and 25 years ($\bar{X}$ = 9.39). According to the playing position, the sample consisted of goalkeepers (*N* = 24), wings (*N* = 44), center backs (*N* = 27), backs (*N* = 48), and pivots (*N* = 27).

### Variable

Sociodemographic variables included: gender (male/female), and age (continues variable). Sport participation variables included: level of sports participation (professional or semi-professional), years of training handball (continues variable), and players’ position (pivot, wing, goalkeeper, back). Mental skills variables included subscales of Bull’s Mental Skills Questionnaire.

### Instruments

The Bull’s Mental Skills Questionnaire was developed in the United Kingdom to measure imagery ability (IA, items 1–4), mental preparation (MP, items 5–8), self-confidence (SC, items 9–12), anxiety and worry management (AWM, items 13–16), concentration ability (CA, items 17–20), relaxation ability (RA, items 21–24) and motivation (M, items 25–28) from which a total scale score is derived (Bull et al., 1996). The questionnaire consists of 28 items and assesses participants along a 6-point Likert scale, requiring item responses ranging from “strongly agree” to “strongly disagree.”

### Procedure

Prior to conducting the study, consent was obtained for the use of The Bull’s Mental Skills Questionnaire, which was then translated into Serbian for the first time. The translation was done by two researchers: one Ph.D. psychologist and one Ph.D. sports researcher. Evaluators have gone through short training about the proper administration of the questionnaire. During 2018 and 2019, data were collected from handball clubs in the territory of Serbia. Respondents were instructed on the goal of the research, and only athletes who expressed a desire to be part of the research participated. All participants were provided with anonymity, and all ethical principles of research were observed. The studies involving human participants were reviewed and approved by the ethical board of the University of Novi Sad, Serbia (Ref. No. 46-06-02/2020-1). Respondents filled out the questionnaire *via* pen and paper in the presence of the evaluator.

### Statistical analysis

The modified exploratory factor analysis was used to determine the latent dimensions of the Bull’s Mental Skills Questionnaire. Namely, as the data of the questionnaire are of the ordinal type, in the first phase, it was necessary to calculate the correlations between all items by Spearman’s procedure, then import the obtained intercorrelation matrix and perform a factor analysis on it. The resulting intercorrelation matrix was then rotated into a more favorable parsimonious promax solution, and the number of significant principal components was determined using multiple criteria (Kaiser–Guttman, parallel analysis, Scree). The most logical solution was taken as acceptable. Differences between male and female handball players in the manifestation of mental skills were obtained by applying the Mann–Whitney *U* test, and the criterion of statistical significance was defined at *p* ≤ 0.05. Using the Kruskal–Wallis one-way analysis of variance, differences depending on playing positions were determined, and the *post hoc* analysis was followed by the Mann–Whitney test with the same boundary criterion of statistical significance. For all these statistical analyses and procedures, IBM SPSS Statistics version 26 was used and software package R ver. 4.1.1 while using the following libraries: psych, corrgram, nFactors, ggplot2.

## Results

### Goal 1: Psychometrics analysis of Bull’s Mental Skills Questionnaire

Tables 2, 3 show the main components of the analyzed questionnaire. Based on the conducted factor analysis of the pattern matrix, five factors were defined in relation to seven that the authors of the questionnaire assume. The first factor consists of items related to the athlete’s ability to concentrate and the intensity of anxiety and worry they experience. This factor is formed by the items in the original setting on the Anxiety and Worry management factor (Items 13, 14, 16) and the Concentration ability factor (items 18, 19) and item 22. Therefore, in this study, this factor is called Anxiety and Concentration management, which consists of 6 items, where a higher score indicates a lack of ability of the athlete to regulate the degree of anxiety and worry, as well as the inability to focus and concentrate on the task ahead. The internal validity of this factor is 0.74, which indicates a satisfactory validity that does not deviate to a greater extent from the results obtained in the sample of students of sports and physical activity in South Africa, United Kingdom, and Turkey (Table 4). The second factor includes the projection of items related to the athlete’s ability to set realistic goals in sports and the belief that he/she can achieve them. Factors that are in the original setting within the Mental preparation (item 5, item 8) and Self-confidence (items 9, 10, 11, 12) factors are projected on this factor. Bearing in mind that item 5 and item 8 describe the belief in the fulfillment of the set goal, their projection of the same factor related to the athlete’s self-confidence represents a logical position. Therefore, this factor is called Self-confidence, and its internal validity is 0.75, which is in line with the validity achieved on various samples outside the territory of Serbia. A higher score indicates a higher level of self-confidence. The third factor consists of three items that relate to the athlete’s ability to relax and maintain an optimal level of emotional arousal. The factor consists of items that refer to the ability to relax on the original factor structure of the questionnaire, whereby item 22, which refers to the pronounced degree of tension before a competition in our sample, is projected on a factor indicating the athlete’s ability to cope with anxiety. Therefore, the third factor is called Relaxation ability and shows a slightly lower internal validity (0.66) in relation to the obtained validity on a sample of African and English students. A higher score indicates the athlete’s ability to relax and maintain an optimal level of emotional arousal. Five items are projected on the fourth factor, of which three items are from the original Motivation factor (items 26, 27, 28) and two from Mental preparation (items 6 and 7). Items 6 and 7 focus on the athlete’s ability to set specific goals and analyze them after successful or unsuccessful realization, while items 26, 27, and 28 refer to the athlete’s ability to get up and activate for competition, which can be seen as a form of mental preparation which is motivational in nature, while items 6 and 7 refer to pre-cognitive mental preparation. Therefore, this factor is called Mental preparation, whose internal validity is 0.68, where higher scores indicate a greater ability to mentally prepare. Three items (items 1, 2, and 4) are projected on the fifth factor, which refers to the athlete’s ability to apply visualization and imagination techniques in order to improve the performance of a certain movement. Items that are projected on this factor and in the original setting make up the Imagery ability factor, so in this study, the fifth factor is called Imagery ability, whose internal validity is 0.66. A higher score indicates a higher degree of visualization and imagination.

**TABLE 2:** Principal components (H), eigenvalues (λ), and percentage of common variance explained (R2)—main components with disturbing ones removed.

Item	H1	H2	H3	H4	H5
mskill1	1.499	2.027	2.218	0.502	1.402
mskill2	1.420	1.754	2.438	–0.107	0.804
mskill4	0.553	1.891	1.932	1.679	–1.449
mskill5	2.046	1.492	–0.340	–1.641	0.141
mskill6	1.078	2.830	–1.610	0.219	0.674
mskill7	1.028	2.411	–1.480	–0.735	–1.411
mskill8	1.553	1.096	0.479	–1.664	–1.732
mskill9	3.082	–2.210	–0.064	–0.769	0.661
mskill10	4.045	–0.890	0.496	–0.432	0.190
mskill11	3.211	–2.001	–0.199	–1.081	0.966
mskill12	3.342	–0.258	–0.442	–0.312	0.637
mskill13	–6.421	0.082	–0.072	–0.262	0.100
mskill14	–5.890	0.095	1.049	0.064	0.409
mskill16	–5.684	0.160	–0.005	–0.570	–0.509
mskill18	–7.135	–1.178	–0.371	0.121	–0.043
mskill19	–7.192	–1.447	0.275	0.162	0.288
mskill21	1.757	–2.563	1.055	–0.040	–0.777
mskill22	–5.586	0.154	–1.432	0.148	0.343
mskill23	3.004	–1.979	0.269	0.519	–0.527
mskill24	2.796	–1.995	–0.071	0.971	–0.950
mskill26	2.423	–0.855	–1.251	1.363	–0.219
mskill27	3.102	0.236	–0.966	0.612	0.565
mskill28	1.968	1.146	–1.911	1.252	0.438
λ	4.20	2.50	2.25	2.36	1.92
*R* ^2^	0.32	0.19	0.17	0.18	0.15

**TABLE 3:** Pattern (A) matrix and communalities (h2).

Item	A1	A2	A3	A4	A5	h^2^
mskill13	0.79	0.11	–0.01	–0.02	–0.02	0.60
mskill18	**0.72**	–0.05	0.05	–0.01	–0.24	0.59
mskill14	**0.72**	0.02	0.04	–0.16	0.24	0.56
mskill19	**0.67**	–0.11	0.06	–0.15	–0.10	0.55
mskill16	**0.67**	0.34	–0.15	–0.06	–0.13	0.54
mskill11	–0.61	**0.52**	–0.12	–0.08	–0.12	0.65
mskill9	–0.52	**0.51**	0.01	–0.11	–0.16	0.59
mskill22	**0.51**	–0.09	–0.17	0.40	–0.20	0.52
mskill8	0.23	**0.67**	0.13	0.03	0.01	0.49
mskill5	0.00	**0.59**	–0.09	0.16	0.10	0.47
mskill10	–0.42	**0.52**	0.09	–0.05	0.07	0.66
mskill12	–0.36	**0.45**	–0.06	0.21	0.00	0.52
mskill24	0.03	–0.06	**0.88**	0.15	–0.09	0.76
mskill23	–0.08	0.02	**0.75**	0.04	0.01	0.65
mskill21	0.06	0.24	**0.71**	–0.27	–0.05	0.57
mskill28	–0.12	–0.06	0.02	**0.77**	0.01	0.62
mskill6	0.08	0.12	–0.13	**0.67**	0.18	0.58
mskill26	–0.17	–0.11	0.38	**0.52**	–0.10	0.51
mskill7	0.17	0.34	–0.03	**0.51**	0.00	0.46
mskill27	–0.13	0.24	0.19	**0.45**	–0.01	0.50
mskill1	–0.17	–0.07	–0.15	0.09	**0.88**	0.76
mskill2	–0.01	0.08	0.01	–0.02	**0.77**	0.65
mskill4	0.24	0.09	0.21	0.11	**0.49**	0.41

Bold values indicate the highest loading (correlation) for each variable (item).

**TABLE 4:** Result of reliability analysis (Cronbach alfa) and comparative review of obtained results in South Africa and United Kingdom (Edwards et al., 2014), Turkey (Miçooğullari et al., 2021), and Serbia.

Factor	South Africa sample (*n* = 211)	United Kingdom sample (*n* = 209)	Turkish sample (*n* = 294)	Serbian sample (*n* = 170)
Imagery ability	0.81	0.44	0.73	0.66
Mental preparation	0.72	0.69	0.52	0.68
Motivation	0.78	0.84	0.64	
Self-confidence	0.70	0.80	0.72	0.75
Anxiety and worry management	0.61	0.66	0.63	0.74
Concentration ability	0.73	0.75	0.71	
Relaxation ability	0.81	0.83	0.69	0.66

Based on the selected factors, the differences in mental skills according to gender, playing position, and the competitive rank were examined.

### Goal 2: Establishing gender differences in the mental skills of handball players

Based on Table 5, it can be seen that statistically significant differences between male and female handball players in mental skills exist only when it comes to Anxiety, Concentration management, and Relaxation ability. Female handball players score higher on Anxiety and Concentration management and lower on Relaxation ability.

**TABLE 5:** Gender differences in mental skills.

Factor	Male handball players (*N* = 99)				Female handball players (*N* = 71)						
	$\bar{X}$ ± SD	Median	IQR	KS	$\bar{X}$ ± SD	Median	IQR	KS	U	Z	*p*
Anxiety and concentration management	13.86 ± 6.21	12	9–20	0.000	15.46 ± 5.60	16	11–19	0.200*	2893.5	−2.0	0.049
Self-confidence	28.98 ± 5.10	30	25–33	0.000	27.87 ± 6.06	29	25–32	0.000	3177.5	−1.1	0.286
Relaxation ability	12.80 ± 3.49	13	10–15	0.006	11.65 ± 3.46	12	9–14	0.200*	2833.0	−2.2	0.031
Mental preparation	23.65 ± 4.17	25	21–27	0.000	24.79 ± 3.93	26	23–27	0.000	2892.5	−1.9	0.061
Imagery ability	12.99 ± 3.62	14	10–16	0.004	13.75 ± 2.89	13	12–16	0.036	3197.0	−1.0	0.313

$\bar{X}$ ± SD, Mean ± Standard Deviation; IQR, Interquartile range; KS, Kolmogorov–Smirnov test; U, Mann Whitney test; Z, Z-value; p, significance. *Statistically significant difference.

### Goal 3: Establishing differences in relation to the playing position in the mental skills of male and female handball players

Playing positions are grouped into goalkeepers, wing, pivotmen, center backs, and backs.

**FIGURE 1:**
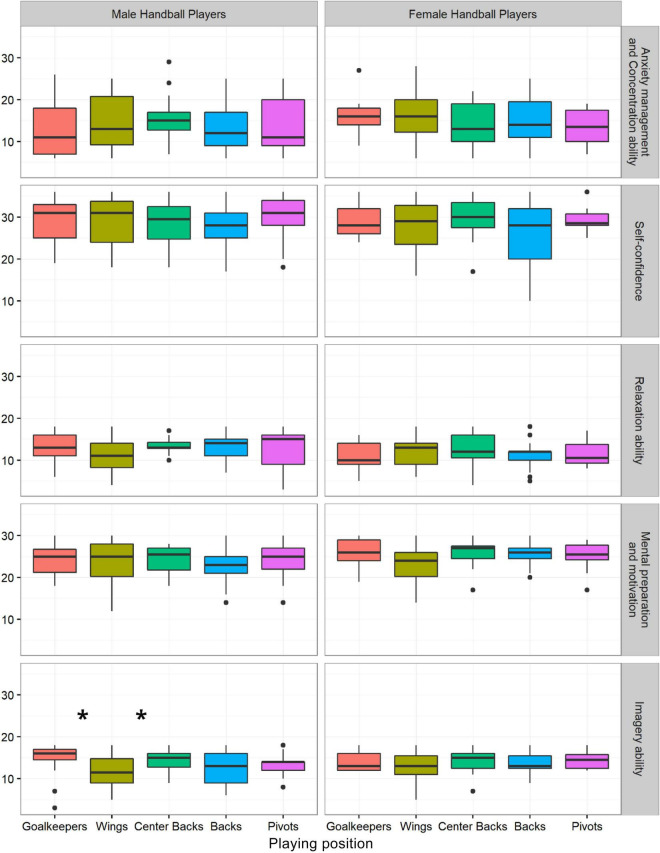
Differences in playing positions in the obtained factors. Data shows medians and interquartile ranges: *p ≤ 0.05.

The results presented in Figure 1, obtained by applying the Kruskal–Wallis one-way analysis of variance, and Mann–Whitney’s *post hoc* analysis. The results suggest that statistically significant differences in playing positions were observed between male goalkeepers and wings (*Z* = 92.00, *p* = 0.020) in the Imagery ability as well as between center backs and wings (*Z* = 104.00, *p* = 0.030). There were no statistical differences among female handball players between the playing positions.

When we look closely at achieved scores on mental skills, even though they do not statistically differ, they contribute to a better understanding of the mental skill of different playing positions. Among male handball players, center backs achieve the highest scores ($\bar{X}$ = 15.56 ± 5.64) on Anxiety and Concentration management, then wings ($\bar{X}$ = 14.27 ± 6.29), pivots ($\bar{X}$ = 13.59 ± 6.59), goalkeepers ($\bar{X}$ = 13.20 ± 7.84), and backs ($\bar{X}$ = 13.10 ± 5.48). When Self-confidence is regard, pivots achieve the highest scores ($\bar{X}$ = 30.12 ± 5.37), then goalkeepers ($\bar{X}$ = 29.47 ± 5.14), wings ($\bar{X}$ = 29.23 ± 5.41), center backs ($\bar{X}$ = 29.06 ± 5.32), and backs ($\bar{X}$ = 27.83 ± 4.67). Center backs achieve the highest average score on Relaxation ability ($\bar{X}$ = 13.56 ± 1.97), then goalkeepers ($\bar{X}$ = 13.27 ± 3.51) and backs ($\bar{X}$ = 13.24 ± 3.02), pivots ($\bar{X}$ = 12.76 ± 4.38), and wings ($\bar{X}$ = 11.36 ± 3.99). Goalkeepers ($\bar{X}$ = 24.29 ± 3.91), center backs ($\bar{X}$ = 24.38 ± 3.48), and pivots ($\bar{X}$ = 24.18 ± 4.14) achieve higher scores on Mental preparation than wings ($\bar{X}$ = 23.45 ± 5.15) and backs ($\bar{X}$ = 22.79 ± 3.95). And finally, in the factor where significant differences were found, goalkeepers ($\bar{X}$ = 14.40 ± 4.19) achieve the highest scores on Imagery ability, then center backs ($\bar{X}$ = 14.13 ± 2.68), pivots ($\bar{X}$ = 13.41 ± 2.72), backs ($\bar{X}$ = 12.48 ± 3.75), and wings ($\bar{X}$ = 11.55 ± 3.88) (Figure 1).

For female handball players, trends are slightly different. Wings achieve the highest score ($\bar{X}$ = 16.73 ± 6.39) on Anxiety and Concertation management, then goalkeepers ($\bar{X}$ = 16.44 ± 4.95), backs ($\bar{X}$ = 15.42 ± 5.56), center backs ($\bar{X}$ = 14.00 ± 5.69), and pivots ($\bar{X}$ = 13.50 ± 4.17). Center backs ($\bar{X}$ = 29.55 ± 5.54), pivots ($\bar{X}$ = 29.50 ± 2.99), and goalkeepers ($\bar{X}$ = 29.11 ± 4.31) achieve higher scores on Self-confidence then wings ($\bar{X}$ = 27.50 ± 5.96) and backs ($\bar{X}$ = 25.89 ± 7.96). The highest score on Relaxation Ability have center backs ($\bar{X}$ = 12.73 ± 4.34), then wings ($\bar{X}$ = 11.82 ± 3.50), pivots ($\bar{X}$ = 11.60 ± 3.24), backs ($\bar{X}$ = 11.16 ± 3.11), and goalkeepers ($\bar{X}$ = 11.00 ± 3.57). Backs ($\bar{X}$ = 25.95 ± 2.88), center backs ($\bar{X}$ = 25.64 ± 3.64), and goalkeepers ($\bar{X}$ = 25.56 ± 4.03) achieve higher scores on Mental preparation, then pivots ($\bar{X}$ = 25.00 ± 3.65) and wings ($\bar{X}$ = 22.95 ± 4.57). And, on the Imagery ability factor, the highest average score achieved pivots ($\bar{X}$ = 14.50 ± 2.12), then goalkeepers ($\bar{X}$ = 14.22 ± 2.49) and center backs ($\bar{X}$ = 14.18 ± 3.25), backs ($\bar{X}$ = 13.63 ± 2.87), and wings ($\bar{X}$ = 13.09 ± 3.25) (Figure 1).

## Discussion

The first goal of the research was to examine the factor structure of the Bull’s Mental Skills Questionnaire (Bull et al., 1996), which has not been tested on a sample of Serbian male and female handball players so far. Compared to the original structure of the questionnaire, which singles out seven factors of mental skills imagery ability (IA), mental preparation (MP), self-confidence (SC), anxiety and worry management (AWM), concentration ability (CA), relaxation ability (RA), and motivation (M), five factors were singled out on the sample of Serbian male and female handball players. The first factor, called Anxiety and Concentration management, consists of six items that are originally positioned on the AWM and CA factors of the original questionnaire. A higher score indicates a lack of ability of the athlete to regulate the degree of anxiety and worry, as well as the inability to focus and concentrate on the task in front of him/her. The second factor, called Self-confidence, consists of six items, which are originally positioned on the SC and MP factors of the original questionnaire. Projecting items from the MP factor to the SC factor makes sense when you take into account that items 5 and 8 refer to the athlete’s belief in the fulfillment of the set goal. A higher score indicates a higher level of self-confidence. The third factor, Relaxation ability, consists of three items that relate to the athlete’s ability to relax and maintain an optimal level of emotional arousal. A higher score indicates a better relaxation ability. The fourth factor, called Mental preparation, consists of five items that are originally positioned on the M and MP factors of the original questionnaire. Guided by the fact that the items relate to the athlete’s ability to set specific goals and analyze them after successful or unsuccessful realization and the ability to raise and activate for competition, the factor is unified by the name “mental preparation”–a type of cognitive and motivational preparation for competition. The fifth factor consists of three items that form the Imagery ability factor in Bull’s questionnaire. A higher score indicates a higher degree of visualization and imagination. The internal validity of the factors proved to be satisfactory and similar to the validity obtained in the sample of English, South African, and Turkish respondents (Table 4).

Regarding gender differences in the mental skills of handball players, the obtained results indicate that female handball players show a lower degree of anxiety regulation ability than male handball players but, on the other hand, show a higher degree of relaxation ability. The obtained results are in line with previous research, which found that female handball players have a somewhat lower ability to regulate emotions and less coping strategies compared to male handball players (Kristjánsdóttir et al., 2018; Ivaskevych et al., 2019; Ökrös et al., 2020). Although the results on gender differences in mental skills are inconsistent, what can be noticed is those female handball players have a higher degree of emotional arousal, which does not have to be a consequence of personality traits, but the situation itself, and that aspects of competitive anxiety and performance concerns are more present with them than with male handball players. The obtained results can be seen from the angle of the sport itself and the sports context. The training process, as well as the attitudes of sports workers toward women’s and men’s sports, can influence the development of sports settings that stimulate sports self-confidence in male handball players and a climate where female handball players lack emotional support necessary for the development of sports confidence (Trbojević et al., 2020). Handball is often seen as a men’s sport, which can contribute to the development of stereotypes and prejudices faced by female handball players, which affect the image of themselves as competent sports persons (Trbojević and Petrović, 2020) and the development of greater concerns about performance. It is evident that sportswomen and sportsmen differ in morphological, motor, and anthropometric characteristics, which may be one of the reasons for the differences in the training process, but one should also keep in mind the social aspect of possible differences.

Playing positions in handball differ, as do the tasks that handball players face in these positions. Thus, a statistically significant difference was obtained in Imagery ability among male handball players, between goalkeepers and wings, and between wings and center backs (Figure 1). Handball players in the center position (center backs or playmakers) and goalkeepers achieve significantly higher scores on imagery ability compared to handball players in the wing position. Center backs are often the leaders of the game, and the largest number of ball exchanges takes place between them, while the wings get the opportunity to shoot in a slightly smaller percentage. This can have an effect on abilities to visual and imagine plays and tactics. Center backs are in charge of game dynamic. They have to see where each teammate and opponent are in order to successfully execute the play. The dynamic of the position of center backs, and wings, could be the reason for obtained differences. The physical position of wings is limited; they do not see the whole playing field from the center as center backs do. A significant difference in this ability was obtained between goalkeepers and wings. Like center backs, the goalkeepers have to have a good attention span and be able to predict where the ball will end up. This dynamic of the playing position can demand more abilities in visualization and imagery—as the closest skills to predicting the next move. The goalkeeper does not just “follows” the ball; he also observes the weak spots in the defense line of his teammates, which can contribute to his heightened abilities to imagine and visualize the play or movement. The position of the goalkeeper requires a high degree of concentration and predictions from which angle the shot will follow. Wings, on the other hand, in the very corner of the field and with a smaller number of ball exchanges, are more focused on the segment of the field than on the big picture, which can affect their ability to imagine.

When it comes to female handball players, significant differences in relation to the playing position were not found (Figure 1). But from achieved scores on five mental skills of each playing position, we can see those wings achieve the highest score on Anxiety and concentration management and the lowest scores on Imagery ability and Mental preparation. According to the obtained results, it can be noticed that the players in the wing position achieve slightly lower results when it comes to mental skills and that they can be the target group with whom it is necessary to work on mental toughness. The wing is a specific playing position that usually requires sudden and quick assessments and actions, and which again entails a smaller percentage of opportunities to shoot and exchange balls, which makes it difficult to practice and develop self-confidence and mental skills such as regulating emotions in stressful situations.

Obtained results are somewhat in line with previous research that shows that playmakers (center backs) score highest on subscales of mental skills directed toward attention and concentration process, goal-setting abilities, and mental preparation (Ökrös et al., 2020). In our sample, male center backs achieve a higher score on these subscales which is in line with Grobbelaar and Eloff (2011), who found that goal-attack players outperformed the other positional groups in team sports. However, center backs also have greater anxiety and concentration regarding their abilities, which is not in line with previous research. These results are not significantly higher for center backs in relation to other playing positions but are important to point out in order to further investigate do male center backs in Serbian handball have greater anxiety, what type is it, and is it a question of cultural and Serbian training process of center backs.

Wings, both in male and female samples of handball players, show slightly lower mental skills. Compared to previous research that found that players in these positions score higher on self-confidence and achievement motivation (Ökrös et al., 2020), the Serbian sample of wings players achieved high scores on anxiety, moderate self-confidence, and the lowest abilities of relaxation and imagination compared to other positions. It can be seen that players in the wing position would benefit most from working on mental skills, as well as players in the center back position in the form of the development of strategies to reduce competitive anxiety. These results invite sports experts to reinvestigate the game tactic and training program in Serbian handball.

## Conclusion

• The internal validity of the factors of the Bull’s Mental Skills Questionnaire proved to be satisfactory and similar to the validity obtained in the sample of English, South African, and Turkish respondents.
• Compared to the original structure of the questionnaire, which singles out seven factors of mental skills (imagery ability, mental preparation, self-confidence, anxiety and worry management, concentration ability, relaxation ability, and motivation), five factors were singled out in the sample of Serbian male and female handball players (anxiety and concentration management; self-confidence; relaxation ability; mental preparation, and imagery ability).
• Male and female handball players differ statistically significantly in the degree of emotional regulation, whereas male handball players show a higher degree of emotional regulation and the ability to concentrate compared to female handball players. Results regarding gender differences in sports are often non-consistent, which is why it is necessary to continue investigating and observing if those differences influence sports achievement and the wellbeing of athletes. In the Serbian sample, obtained differences may be the result of the social aspect of handball.
• Male handball players differ in Imagery ability in relation to the player’s position. A significant difference was obtained between the wing players and goalkeepers and wings and center backs. Players in the wing position achieve the lowest score on Imagery ability. Significant differences in relation to playing position were not obtained in female handball players. These results indicate the need to work with players in the wing position on the development of mental skills (reduction of emotional arousal, relaxation, and imagination skills); and with players in the center backs position to develop strategies to reduce competitive anxiety. Also, the result indicates the need to work with all-female players on the development of mental skills aimed at emotional regulation, especially with female players in the wing position, in order to improve emotional regulation.
• Obtained results invite further investigation of handball players mental skills in relation to age, level of sports participation, and interaction between mental skills and other individuals (personality traits, goal orientation, motivation) and situational factors (exp. motivational climate, sport achievement, type of coaching…).
• In line with the understanding that mental skills can be taught, further research should focus on investigating the effects of mental training programs for handball players.

## Data availability statement

The original contributions presented in this study are included in the article/supplementary material, further inquiries can be directed to the corresponding author.

## Ethics statement

The studies involving human participants were reviewed and approved by Ethical Board of the University of Novi Sad, Serbia (Ref. No. 46-06-02/2020-1). Written informed consent to participate in this study was provided by the participants’ legal guardian/next of kin.

## Author contributions

DJ and JT contributed to the experimental design, data collection and analysis, and drafting of the manuscript. BM contributed to the experimental design and revision of the manuscript. SM and NF contributed to the data collection and examined the study. DS, AB, and PD conceived of and examined the study and revised the manuscript. All authors contributed to the article and approved the submitted version.

## Footnotes

1 Semi-elite athletes are those whose highest level of participation is below the top standard possible in their sport (e.g., in talent-development programs, competing at second-tier standard or below, etc.); competitive-elite athletes regularly compete at the highest level in their sport (e.g., top divisions/leagues) but have not had any success at that level; successful-elite athletes compete at the highest level, and have experienced some (infrequent) success at that standard (e.g., winning an event or a medal) (Swann et al., 2015).
